# Neutrophil dysmorphisms and extracellular trap-like structures in a patient with severe coronavirus disease 2019

**DOI:** 10.1590/0037-8682-0099-2026

**Published:** 2026-06-15

**Authors:** Andrezza do Espirito Santo Cucinelli, Diêgo Roberto dos Santos, Hye Chung Kang, Isabela Resende Pereira

**Affiliations:** 1 Universidade Federal Fluminense, Faculdade de Medicina, Departamento de Patologia, Niterói, RJ, Brasil.

Severe coronavirus disease 2019 (COVID-19) is associated with notable changes in circulating neutrophils. Examination of a peripheral blood smear from a patient with severe illness revealed significant abnormalities in neutrophils, indicating intense inflammatory activation (Figure 1).

**FIGURE 1:**
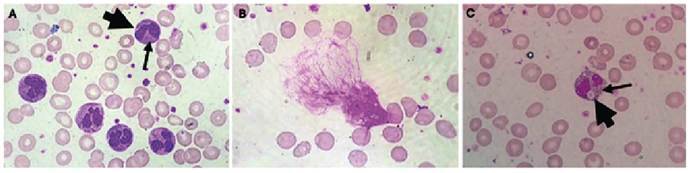
Peripheral blood smear showing neutrophil dysmorphisms in a patient with severe coronavirus disease 2019. May-Grünwald-Giemsa-stained peripheral blood smear (original magnification ×400). **(A)** Segmented neutrophils with marked toxic granulation (small arrow); the larger arrow indicates a bilobed (Pelger-Huët-like) neutrophil. **(B)** Extracellular mesh-like structure indicative of chromatin release during neutrophil activation (neutrophil-extracellular-trap-like structure). **(C)** Hyposegmented neutrophil with toxic granulation (small arrow) and an intracytoplasmic vacuole (larger arrow), consistent with intense innate immune activation.

May-Grünwald-Giemsa-stained smears revealed prominent neutrophil dysmorphisms. Panel A shows segmented neutrophils with marked toxic granulation and nuclear hyposegmentation, including bilobed (Pelger-Huët-like) forms. Similar abnormalities have been described in severe infections, and they are linked to dysregulated myelopoiesis^1^ ^,^ ^2^. Panel B shows an extracellular mesh-like structure indicative of chromatin release during neutrophil extracellular trap (NET) formation. Excessive NETosis has been implicated in hyperinflammation and immunothrombosis in patients with severe COVID-19^3^ ^,^ ^4^. Panel C shows a hyposegmented neutrophil with toxic granulation and an intracytoplasmic vacuole, indicating active phagocytosis.

Circulating neutrophil dysmorphisms, including toxic granulation, hyposegmentation, and extracellular chromatin release, have been consistently reported in patients with a severe viral infection. The dysmorphisms correlate with systemic inflammation and poorer clinical outcomes^4^ ^-^ ^6^. These images illustrate how examination of routine peripheral blood smears can reveal morphological patterns associated with severe inflammatory activation, emphasizing the ongoing importance of microscopic evaluation in infectious diseases.

## Data Availability

Research data is available in the body of the article (Results).
